# Effectiveness of Early Advanced Glycation End Product Accumulation Testing in the Diagnosis of Diabetes: A Health Risk Factor Analysis Using the Body Mass Index as a Moderator

**DOI:** 10.3389/fendo.2021.766778

**Published:** 2022-03-11

**Authors:** Yi Zhang, Tian Jiang, Chao Liu, Honglin Hu, Fang Dai, Li Xia, Qiu Zhang

**Affiliations:** ^1^ Department of Endocrinology and Metabolism, The First Affiliated Hospital of Anhui Medical University, Hefei, China; ^2^ Department of Maternal, Child and Adolescent Health, School of Public Health, Anhui Medical University, Hefei, China

**Keywords:** advanced glycation end products, BMI, diabetes, age, prediabetes, risk factors, early screening

## Abstract

**Objective:**

To evaluate the value of non-invasive detection of advanced glycation end products (AGEs) in the early screening of type 2 diabetes mellitus (T2DM) in the community of China.

**Methods:**

From January 2018 to January 2019, a total of 912 patients with community health physical examination and no history of T2DM were selected, excluding the results of missing value > 5%. Finally, 906 samples were included in the study, with a response rate of 99.3%. Non-invasive diabetic detection technology was used to detect AGEs in the upper arm skin of all participants, AGE accumulations were classified as ≤P25, P25∼P50, P50∼P75, and >P75; HbA1c, insulin, C-peptide, total cholesterol (TC), triglyceride (TG), high-density lipoprotein cholesterol (HDL-C), creatinine, urea, and other indicators were measured at the same time. Univariate analysis of variance was used to compare the differences in general data, biochemical indexes, skin AGE levels, and blood glucose among groups, and logistic regression analysis and latent category analysis were performed.

**Results:**

In univariate analysis, SBP, FBG, HbA1c, and age were correlated with higher AGE (p < 0.01); TG, TC, HDL, UA, and gender were not positively correlated with AGE (p < 0.01). After controlling for covariates (waist circumference, hip circumference), AGE accumulation was interacted with other variables. The results of latent category analysis (LCA) showed that the health risk factors (HRFs), including age, systolic blood pressure, HbA1c, FBG, triglyceride, total cholesterol, HDL-C, and uric acid, were divided as three groups, and AGE is divided into four categories according to the quartile method, which were low risk (≤P25), low to medium risk (P25∼P50), medium to high (P50∼P75), and high risk (>P75), respectively. The association between the quartile AGE and risk factors of the OR values was 1.09 (95% CI: 1.42, 2.86), 2.61 (95% CI: 1.11, 6.14), and 5.41 (95% CI: 2.42, 12.07), respectively. The moderation analysis using the PROCESS program was used to analyze whether BMI moderated the link between risk factors and AGE accumulation. There was also a significant three-way interaction among HRFs, BMI, and gender for AGE accumulation in the total sample (β = -0.30).

**Conclusion:**

Non-invasive skin detection of AGEs has a certain application value for the assessment of T2DM risk and is related to a variety of risk factors.

## Introduction

About one in 10 middle-aged Europeans will develop type 2 diabetes over a 10-year period (1). This number may be due to the increased threat of the pandemic, and part of the reason is the increase in obesity; it is predicted that by 2030, the prevalence of type 2 diabetes will reach 366 million people around the world (www.who.int/diabetes/facts/world_figures/en/) (2). Prediabetes, also known as impaired glucose regulation, is a metabolic state between normal glucose tolerance and diabetes. In a previous nationally representative cross-sectional survey conducted in Mainland China in 2013, a total of 170,287 people participated, and the estimated prevalence of diabetes was 10.9% and that of prediabetes was 35.7% (3). Therefore, early diagnosis and intervention of prediabetes are of great significance. In addition, there is increasing evidence that the concept of "metabolic memory" was associated with the development of long-term metabolic-related disorders and plays a significant role in patients with diabetes (4). The existing main metabolic pathways including oxidative stress, inflammation, and advanced glycation end products (AGEs) play an important role in the pathogenesis of late diabetic complications, and the measurement of AGEs appears to be the prospective significantly appropriate method to evaluate the true impact of chronic hyperglycemia (5). Previous study have shown that there is increased expression of AGEs and their accumulation in the diabetes mellitus (DM) tissues (6). Many risk factors are known, but all factors taken together do not fully explain the risk of diabetes complications. This suggests that other pathophysiological mechanisms are at work. An increase in tissue AGEs may be a surrogate mechanism. As a non-invasive clinical tool, skin autofluorescence can be used to assess the accumulation of AGEs (7, 8). More and more evidence shows that AGEs can cause mitochondrial dysfunction of pancreatic islets, resulting in excessive superoxide production, reduced ATP level, and ultimately insulin secretion dysfunction, leading to continuous hyperglycemia in the body (9). AGEs bind to RAGE (AGE receptor), leading to mitochondrial swelling and apoptosis, and impaired glucose aerobic oxidation, further promoting the formation of AGEs (10, 11). Zhu et al. developed a non-invasive diabetes detector (DM Scan) by applying the optical detection technology of AGEs and confirmed that AGEs on the skin, as a screening indicator for diabetes, has the advantages of being rapid, non-invasive, and consumable and having no risk of cross infection (12). In addition, AGEs can also induce pancreatic beta cell apoptosis by inhibiting the autophagy of beta cells. Moreover, AGEs can not only directly damage islet β cells, which is related to the occurrence and development of diabetes, but also accumulate in almost any tissue, including the kidneys, eyes, liver, reproductive tissue, vascular system, muscle, bone, and brain (13). Besides, increased AGE levels have been associated with many microvascular diabetic complications (14), further explaining why we explore the association between metabolic index and AGE and newly diagnosed diabetic patients and prediabetes.

At present, clinical management of diabetes mainly focuses on blood glucose control, blood pressure control, lipid lowering, lifestyle, and other risk factors. If blood glucose is a major factor in the occurrence of these complications, then by normalizing blood glucose levels (15), diabetes can be cured and complications can be prevented by 100%. These studies suggest that glycemic control has a long-term impact on the development and progression of diabetes complications. In addition, all eligible studies showed a positive association between skin autofluorescence (SAF) and one or more diabetes complications (all-cause mortality, cardiovascular mortality, microvascular and macrovascular complications, neuropathy, and kidney disease) (7). At present, a large-sample invasive serological examination is difficult to be popularized in some grassroots and communities, and there is still a lack of relevant reports on whether non-invasive skin detection of AGEs is suitable for early risk screening of diabetes. This non-invasive method uses skin autofluorescence (SAF) and is based on the specific fluorescence characteristics of some AGEs. Therefore, the author aims to explore the application value of the non-invasive diabetes detector in the early screening of large-scale diabetes in the Chinese population by extensively carrying out the non-invasive and rapid skin detection project of AGEs in the community.

AGEs can cause islet mitochondrial dysfunction, resulting in excessive superoxide production and reduced ATP level, and eventually lead to insulin secretion dysfunction, leaving the body in a state of continuous hyperglycemia. After AGEs and RAGE (receptors of AGE) were combined, the expression of the Bcl-2 gene in islet β cells was decreased, leading to mitochondrial swelling and apoptosis, and impaired glucose aerobic oxidation, which further enhanced the formation of AGEs. In addition, AGEs can also induce pancreatic beta cell apoptosis by inhibiting the autophagy of beta cells, and AGEs can directly damage islet β cells, which is related to the occurrence and development of type 2 diabetes mellitus (T2DM). Moreover, AGEs and their receptors may play important roles in the development and progression of coronary artery disease (CAD) in patients with T2DM (16). Moreover, the AGE-1-positive patients had higher triglycerides and lower high-density lipoprotein (HDL) cholesterol (17).

At present, a large-sample invasive serological examination is difficult to be popularized in some grassroots and communities, and there is still a lack of relevant reports on whether non-invasive skin detection of AGEs is suitable for early risk screening of T2DM. Therefore, the author aims to explore the application value of the non-invasive T2DM detector in the early screening of large-scale T2DM in the Chinese population by extensively carrying out the non-invasive and rapid skin detection project of AGEs in the community.

## Samples and Methods

### Samples

Using the multistage sampling method, from January 2018 to January 2019, three community health service centers in Hefei that signed the medical consortium agreement with the First Affiliated Hospital of Anhui Medical University were selected to screen the population aged 30 to 80 years in each community. The exclusion criteria are as follows: previous history of T2DM, severe cardiovascular and cerebrovascular diseases, liver and kidney function impairment, infection or stress, and people in the terminal stage of various diseases.

### Methods

Doctors and personnel with professional training in designated physical examination institutions had undergone physical checkups based on standard procedures, such as height, weight, AGE test, fundus examination, blood pressure, and blood and urine samples.

Gender, age, height, weight, waist circumference, hip circumference, blood pressure, and other information of patients in all participants were recorded, and the body mass index (BMI) and waist–hip ratio (WHR) were calculated. Fasting venous blood was collected, and measured fast plasma glucose (FPG), 2h plasma glucose (2hPG), glycated hemoglobin A1c (HbA1c), insulin, and C-peptide were tested by the oral glucose tolerance test (OGTT); total cholesterol (TC), triglyceride (TG), high-density lipoprotein (HDL-C), creatinine, urea, and other indicators were tested by blood serum. These risk factors are collectively referred to as health risk factors (HRFs). At the same time, the non-invasive T2DM detector (National Medical Device Registration Certificate: Anhui Injection Certificate 20152210045) was used to detect the fluorescence of the subjects’ upper arm skin. Obvious blood vessels, scars, and malformed skin areas should be avoided during measurement. Selected test sites should be wiped with alcohol to obtain the fluorescence spectrum intensity of AGEs on subjects’ skin (unit: AU). Measurements should be made for 3 times, and the average value should be taken as the final skin AGE testing results. The ethics committee approval number is PJ2019-09-05.

### Criteria for Risk Factors

Hypertension was characterized *via* systolic blood pressure (SBP) ≥ 140 mmHg and/or diastolic blood pressure (DBP) ≥ 90 mmHg (WHO 1999 criteria) (18) or currently using medications for the lowering of blood pressure. Hyperlipidemia was characterized *via* serum concentrations of TC>5.69 mmol/l or TG>1.68 mmol/l or HDL>1.03 mmol/l or patients that have been treated with medications for lowering of lipids. Overweight was defined as a BMI >24 and <28 kg/m^2^. Obesity was characterized as BMI ≥ 28 kg/m^2^ based on Chinese standards (19). An elevated level of HbA1c was greater than 7.0% (20). Hyperuricemia was characterized *via* serum uric acid level ≥ 416 mmol/l (≥7.0 mg/dl) in men and ≥386 mmol/l (≥6.5 mg/dl) in women or if they were treated with allopurinol for lowering the level of uric acid (21). The age limit was 65 years (22). FBG (fast blood glucose) was ≥7.0 mmol/l (23, 24).

### Sensitivity Analysis

In order to improve the effect of HRFs results, we used three different approaches to operationalize HRFs. First, we calculate a HRFs score which added together the total number of HRFs experienced by each participant. This was then categorized as 0 HRFs, 1 HRF, 2 HRFs, and 3+ HRFs. Second, factor analysis (FA) was used to derive distinct variable centered HRFs clusters, namely groups of HRFs that tend to co-occur across participants. This approach helped us to reduce the risk of overfitting. In the analysis, each FA-derived cluster was indexed by a binary variable indicating the presence or absence of any exposure to the relevant HRFs throughout childhood. Third, latent class analysis (LCA) was performed using Mplus in order to derive HRFs clusters.

### Statistical Analysis

All the data were analyzed using SPSS 23.0. Statistical analyses were carried out in three main steps. Missing data on the outcome and exposure variables were estimated using multiple imputations by SPSS 23.0. Latent class analysis (LCA) was used to identify homogeneous, mutually exclusive “patterns” of 6 HRFs using MPlus 7.4. Model building begins with class enumeration, and with the addition of each subsequent latent class, goodness-of-fit indices are compared. The HRF classes were adopted based on model fit indices: Akaike information criteria (AIC), Bayesian information criteria (BIC), and sample size-adjusted BIC (aBIC), entropy, and p-value for the Lo–Mendell–Rubin test (LMRT).

The specific statistical analyses were carried out in four main steps. Step 1 entailed the descriptive statistics associated with the health risk factor by AGE accumulation. In Step 2, multilevel logistic regression was used to test the association between risk factors and AGEs. In Step 3, according to the principle of LCA, the Mplus program was used to perform LCA. In Step 4, the PROCESS program of moderation was used to perform a moderation analysis (25). For testing the moderating effect, the relationships of the three small steps had to be significant: (a) direct effect of predictor (health risk factors) on AGE accumulation; (b) direct effect of moderator (BMI) on AGE accumulation; and (c) direct interactions effect (health risk factors × BMI) on AGE accumulation. In the SPSS PROCESS, the interacting effect was calculated automatically by the program, and it also produces the proportion of the variance explained by the moderating effect of BMI (R2 increases owing to interaction). Considering the influence of covariates, we also adjusted the influence of relevant sociodemographic factors in the adjustment model. We also explored whether BMI moderated the relationship between HRFs and AGE accumulation by gender (26). According to statistical requirements, the moderating effect is considered significant when the 95% CI interval does not contain zero (27).

## Results

### The Prevalence Characteristics of AGE Accumulation

**Table 1** presents the results of the risk factor and AGE accumulation; these results show that higher HbA1c (*χ* ^2^ = 25.76), FBG (*χ* ^2^ = 37.38), SBP (*χ* ^2^ = 8.46), and older age (*χ* ^2^ = 231.11) were more likely to have a higher prevalence of AGE accumulation; others such as HDL (*χ* ^2^ = 4.29), UA (*χ* ^2^ = 2.71), TC (*χ* ^2^ = 6.24), TG (*χ* ^2^ = 3.90), and gender (*χ* ^2^ = 1.94) have no correlation with AGE accumulation.

**Table 1:** The prevalence characteristics of AGEs.

	AGE (≤P25)	AGE (P25～P50)	AGE (P50～P75)	AGE (> P75)	*χ* ^2^ value
HbA1c					25.76^**^
Normal	218 (26.50%)	212 (25.70%)	207 (25.10%)	187 (22.70%)	
Abnormal	10 (12.20%)	13 (15.90%)	21 (25.60%)	38 (46.30%)	
HDL					4.29
Normal	216 (25.80%)	204 (24.40%)	206 (24.60%)	210 (25.10%)	
Abnormal	12 (17.10%)	21 (30.00%)	22 (31.40%)	15 (21.40%)	
UA					
Normal	161 (24.00%)	168 (25.00%)	169 (25.10%)	174 (25.90%)	2.71
Abnormal	67 (28.60%)	57 (24.40%)	59 (25.20%)	51 (21.80%)	
FBG					37.38^**^
Normal	216 (26.50%)	214 (26.30%)	204 (25.10%)	180 (22.10%)	
Abnormal	12 (13.30%)	11 (12.20%)	22 (24.40%)	45 (50.00%)	
TC					6.24
Normal	203 (26.00%)	200 (25.60%)	190 (24.40%)	187 (24.00%)	
Abnormal	25 (19.80%)	25 (19.80%)	38 (30.20%)	38 (30.20%)	
TG					3.90
Normal	164 (25.90%)	165 (26.10%)	155 (24.50%)	148 (23.40%)	
Abnormal	64 (23.40%)	60 (21.90%)	73 (26.60%)	77 (28.10%)	
SBP					8.46^*^
Normal	187 (27.00%)	177 (25.60%)	166 (24.00%)	162 (23.40%)	
Abnormal	40 (19.10%)	47 (22.50%)	61 (29.20%)	61 (29.20%)	
Age					231.11^**^
<65	215 (32.90%)	195 (29.90%)	164 (25.10%)	79 (12.10%)	
≥65	12 (5.00%)	30 (12.40%)	58 (24.00%)	142 (58.70%)	
Gender					1.94
Male	124 (25.40%)	115 (23.60%)	120 (24.60%)	129 (26.40%)	
Female	104 (24.90%)	110 (26.30%)	108 (25.80%)	96 (23.00%)	

*p ＜ 0.05, **p ＜ 0.01.

### The Multilevel Logistic Regression Between Risk Factors and AGE

The demographic data of all participants (gender, age, nationality), physical examination (systolic pressure, diastolic blood pressure, BMI), T2DM number and type of glycemic index (fasting plasma glucose, 2 h postprandial blood glucose, glycosylated hemoglobin), renal function, blood urea nitrogen, serum creatinine, blood uric acid, and blood lipid (total cholesterol, triglycerides, high-density lipoprotein cholesterol (HDL-c)) are shown in **Table 2**; according to the multivariate variable analysis, the AGE accumulation was divided into ≤P25, P25~P50, P50~P75, and >P75 according to the quartile method. SBP, FBG, HbA1c, and age were positively correlated with AGE (OR = 0.621, 95% CI: 0.378, 0.953), (OR = 0.239, 95% CI: 0.121, 0.47), (OR = 0.243, 95% CI: 0.116, 0.507), and (OR = 0.021, 95% CI: 0.01, 0.042), respectively; TG, TC, HDL, and UA were not positively correlated with AGE (OR = 0.239, 95% CI: 0.121, 0.47), (OR = 0.239, 95% CI: 0.121, 0.47), (OR = 0.787, 95% CI: 0.357, 1.736), (OR = 0.239, 95% CI: 0.121, 0.47), and gender (OR = 1.042, 95% CI: 0.706, 1.539). These results are shown in **Table 2**.

**Table 2:** The multilevel logistic regression between risk factors and AGE.

	AGE (≤P25)	AGE (P25～P50)	AGE (P50～P75)	AGE (>P75)
HbA1c				
Normal	0.243 (0.116, 0.507)^**^	0.44 (0.2, 0.967)^*^	0.682 (0.29, 1.603)	1.0
Abnormal	1.0	1.0	1.0	1.0
HDL				
Normal	0.787 (0.357, 1.736)	0.516 (0.247, 1.078)	0.528 (0.252, 1.107)	1.0
Abnormal	1.0	1.0	1.0	1.0
UA				
Normal	1.386 (0.903, 2.128)	1.132 (0.747, 1.715)	1.166 (0.768, 1.769)	1.0
Abnormal	1.0	1.0	1.0	1.0
FBG				
Normal	0.239 (0.121, 0.47)^**^	0.5 (0.239, 1.046)	1.101 (0.462, 2.621)	1.0
Abnormal	1.0	1.0	1.0	1.0
TC				
Normal	0.621 (0.358, 1.076)	0.642 (0.371, 1.111)	1.008 (0.557, 1.824)	1.0
Abnormal	1.0	1.0	1.0	1.0
TG				
Normal	0.952 (0.644, 1.406)	1.022 (0.693, 1.508)	1.046 (0.709, 1.543)	1.0
Abnormal	1.0	1.0	1.0	1.0
SBP				
Normal	0.6 (0.378, 0.953)^**^	0.56 (0.352, 0.889)^**^	0.73 (0.45, 1.19)	1.0
Abnormal	1.0	1.0	1.0	1.0
Age				
<60	0.021 (0.01, 0.042)^**^	0.131 (0.066, 0.26)^**^	0.323 (0.157, 0.668)^**^	1.0
>60	1.0	1.0	1.0	1.0
Gender				
Male	1.042 (0.706, 1.539)	0.969 (0.657, 1.427)	0.957 (0.648, 1.411)	1.0
Female	1.0	1.0	1.0	1.0

*P ＜ 0.05, **P ＜ 0.01.

### Class Enumeration

**Table 3** depicts the fit statistics for the one to four class models. The five-class model did not replicate the best log likelihood value and was therefore not considered further. The four-class solution was chosen as the final, best-fitting model based on the lower AIC, as well as lowest BIC and aBIC values. Moreover, the p value of LMR was not statistically significant in class 4. Moreover, we listed the trend of aBIC values and found that class three has the most significantly descending aBIC values. With regard to model 3, bootstrap validation procedures also demonstrated that it had a good fit (*p* < 0.01). All remaining results are reported specific to the three-class solution.

**Table 3:** The latent class analysis of risk factors.

Statistic	2 classes	3 classes	4 classes
AIC	5312.78	5234.38	5227.63
BIC	5384.91	5344.98	5376.71
aBIC	5337.28	5271.94	5278.26
LMR-LRT	<0.001	0.1239	0.2856
BLRT	<0.001	<0.001	<0.0697
Entropy	0.905	0.687	0.749

As estimated from the model posterior probabilities, there were approximately 42.4%, 9.2%, and 49.2% of participants distributed across the four classes, respectively.

### The Multilevel Logistic Regression of Risk Factors and AGE and BMI

As shown in **Table 4**, all risk factor patterns were statistically significant with AGE accumulation of participants. AGE was taken as the dependent variable, with medium risk (OR: 1.50, 95% CI = 1.02, 2.32) and high risk (OR: 5.41, 95% CI = 2.42, 12.07). The results of adjusting co-variables are still meaningful, as shown in **Table 4**.

**Table 4:** The multilevel logistic regression of risk factors and AGE.

	AGE (≤P25)	AGE (P25～P50)	AGE (P50～P75)	AGE (>P75)
Low risk	1.0	1.0	1.0	1.0
Medium risk	1.0	0.96 (0.63, 1.463)	1.54 (1.02, 2.32)^*^	1.50 (0.98, 2.31)
High risk	1.0	1.09 (1.42, 2.86)^**^	2.61 (1.11, 6.14)^*^	5.41 (2.42, 12.07)^**^

Controlled for gender, waist circumference, and hip circumference.

^*^p ＜ 0.05, ^**^p ＜ 0.01.

In the model adjusted for gender, waist circumference, and hip circumference, children exposed to 3+ HRFs had significantly higher AGE levels than those who did not have any HRF (OR: 9.21, 95% CI = 4.74, 17.91). These datasets are shown in **Table 5**.

**Table 5:** The multilevel logistic regression of number health risk factors and AGE.

	AGE (≤P25)	AGE (P25～P50)	AGE (P50～P75)	AGE (>P75)
0	1.0	1.0	1.0	1.0
1	1.0	1.03 (0.65, 1.63)	1.004 (0.62, 1.63)	1.99 (1.16, 3.42)^*^
2	1.0	1.25 (0.71, 2.80)	1.64 (0.92, 2.91)	3.57 (1.94, 6.57)^**^
3+	1.0	1.88 (0.98, 3.61)	4.17 (2.23, 7.82)^**^	9.21 (4.74, 17.91)^**^

Model 1: crude model; Model 2: controlled for gender, waist circumference, hip circumference.

^*^p ＜ 0.05, ^**^p ＜ 0.01.

The statistical results of the EFA of HRFs in the training dataset are shown in **Table 6**. All factors had good discriminant validity since their correlations were lower than 0.85.

**Table 6:** LCA-derived HRF clusters.

	1	2	3
HbA1c	**0.881**	-0.032	-0.026
FBG	**0.88**	0.019	-0.031
TG	0.026	**0.793**	0.022
TC	-0.003	**0.739**	0.037
HDL	0.133	0.264	**-0.746**
Age	0.234	0.234	**0.65**
SBP	0.321	0.188	**0.363**
UA	-0.026	0.009	**0.151**

Bold coefficients indicate the highest factor loading of every item.

In **Table 7**, all HRF patterns were statistically significant with the BMI of participants by gender. Among males, BMI was taken as the dependent variable, with medium risk (OR: 2.81, 95% CI = 1.82, 4.34) and high risk (OR: 2.06, 95% CI = 1.05, 4.06) of overweight. Among females, BMI was taken as the dependent variable, with medium risk (OR: 0.90, 95% CI = 0.39, 2.08) and high risk (OR: 6.02, 95% CI = 2.42,14.99) of obesity. The results of adjusting co-variables are still significant, as shown in **Table 7**.

**Table 7:** The multilevel logistic regression of risk factors and BMI by gender.

	Male	Overweight	Female	Overweight
	Obesity		Obesity	
Low risk	1.0	1.0	1.0	1.0
Medium risk	3.22 (1.69, 6.13)^**^	2.81 (1.82, 4.34)^**^	0.90 (0.39, 2.08)	1.36 (0.87, 2.13)
High risk	0.37 (0.05, 2.91)	2.06 (1.05, 4.06)^*^	6.02 (2.42, 14.99)^**^	0.89 (0.33, 2.39)

Controlled for gender, waist circumference, and hip circumference.

^*^p＜0.05, ^**^p＜0.01.

### Moderation Analysis

Moderation analyses were performed with waist circumference and hip circumference as the control variables. The results are presented in **Table 8**. First, HRFs significantly predicted the severity of BMI (β = -0.13), and gender was not associated with BMI (β = -0.065). Secondly, HRFs significantly predicted the severity of BMI (β = 0.29), and gender was also associated with BMI (β = 0.56). There were also a significant effect between gender and HRFs on AGE (β = -0.26) and BMI (β = 0.073).

**Table 8:** Model characteristics for the moderation analysis.

	BMI model 1			AGE model 2		
	B	t value	*p* value	B	t value	*p* value
HRFs	-0.13	-2.27	<0.05	0.29	2.30	<0.05
Gender	-0.065	-0.73	>0.05	0.56	2.81	<0.01
Int	0.073	2.05	<0.05	-0.26	-3.22	<0.01
R^2^	0.0025			0.0112		
F	4.19			10.40		

Mediate variables: BMI, independent variables: HRFs, dependent variables: AGE.

^*^p ＜ 0.05, ^**^p ＜ 0.01.

Int: HRFs × gender.

The model was controlled for waist circumference and hip circumference.

As shown in **Table 9**, there was also a significant three-way interaction among HRFs, BMI, and gender for AGE accumulation in the total sample (β = -0.30); after controlling for waist circumference and hip circumference, these results were also significant (β = -0.31).

**Table 9:** Model characteristics for the moderation analysis.

	Model 1			Model 2		
	*B*	*t* value	*p* value	*B*	*t* value	*p* value
HRFs	-0.15	-0.82	>0.05	-0.14	-0.76	>0.05
BMI	-1.77	-3.84	<0.01	-1.92	-4.19	<0.01
Gender	-0.25	-0.90	>0.05	-0.20	3.53	<0.01
Int 1	0.47	2.50	<0.05	0.49	2.58	<0.01
Int 2	-0.03	-0.23	>0.05	-0.009	-0.081	>0.05
Int 3	1.03	3.41	<0.01	1.06	3.54	<0.01
Int 4	-0.30	2.40	<0.05	-0.31	-2.53	<0.01
R^2^	0.0061			0.0067		
F	5.75			6.396		

Model 1: crude model, model 2: the model was controlled for waist circumference, hip circumference. Independent variables: HRFs, dependent variables: AGE.

^*^p ＜ 0.05, ^**^p ＜ 0.01.

Int 1: HRFs × BMI; Int 2: HRFs × gender; Int 3: gender × BMI; Int 4: HRFs × gender × BMI.

## Discussion

The earlier discussion evaluates the value of noninvasive detection method to prevent the development of the disease and shows the effectiveness of targeted prevention, including through the establishment of variables based on the genetic risk of equations to evaluate the risk of diabetes and prediabetes, and guide therapy, which reduces blood pressure, body weight, and cholesterol (28). AGEs accumulate in the body as we age; thus, this approach is necessary to provide some preventive measures for elderly patients with more complications. Moreover, the explanation for this accumulation process is accelerated in some cases with glycemic and oxidative stress, leading to higher AGE levels in, for example, patients with diabetes, patients with renal failure, ICU patients, and smokers (29). In general, AGE levels do seem to be strongly correlated with blood level and other metabolic markers. The association of AGEs with TG, TC, and SBP levels were associated with diabetic macrovascular complications that have been reported in previous studies. The explanation for the increased concentration of AGEs in patients with diabetes is that higher HbA1c will disrupt normal glucose metabolism in the muscle and fat cells, leading to insulin-mediated glucose uptake and the circulating process of hyperglycemia (30). Several other studies have shown a link between elevated AGE levels and cardiovascular disease in people with diabetes. SAF is also associated with macrovascular complications in patients with type 2 diabetes (31). In addition, the advantage of AGEs in this study is that AGEs are associated with long-term metabolic memory and their assessment takes into account cumulative glucose exposure and glucose variability, thus overcoming the limitations of HbA1c as a prognostic biomarker for diabetes (32). So AGEs can not only predict diabetes and its complications, but may also be a cause of those complications. To investigate this, intervention trials of drugs that reduce AGE accumulation are needed.

With the development of economy, the prevalence rate of type 2 diabetes is increasing year by year, this disease should be brought into the scope of policy for key prevention and control of chronic diseases, and in the hope that prevention and control mark the service in basic hospitals, such as level 3 first-class hospitals, and incorporate the same in the community hospitals. However, because of T2DM knowledge is not enough, limited community hospital and patient factors such as fear traumatic examination, blood glucose monitoring still cannot spread, leading to low diagnostic rate and population. Therefore, it is necessary to strengthen basic diabetes screening, such as non-invasive AGE testing. In recent years, a large number of studies have confirmed that AGE is one of the important mechanisms for the occurrence and development of chronic complications of T2DM. Studies have found that excessive AGE is one of the important mechanisms leading to type 2 T2DM and its chronic complications (30). The accumulation of AGEs in tissues is a long-term process with a low turnover rate, which may form the substrate of “blood sugar memory” (31). If targeted detection can be carried out, it may be helpful to identify high glucose status and screen out patients at high risk of T2DM. The T2DM non-invasive detector uses the fluorescence characteristics of AGEs to illuminate the excitation light source on the skin tissue of the subjects through the probe, and then obtains the level of AGEs through the fluorescence spectrum information analysis.

Our analysis revealed several key findings. People at potential risk of disease exposed to three or more HRFs had higher AGE accumulation, which further risks toward T2DM and its complication. Among different types and clusters of HRFs, HbAIc and FBG had the largest associations with AGE accumulation. BMI was also associated with higher AGE accumulation among participants exposed to multiple HRFs. Further, the moderation analysis indicated that gender, BMI, and HRFs were associated with AGE accumulation exposure. The relationships among HRFs, BMI, and AGE accumulation generally had an interaction effect.

### Correlations Between Health Risk Factors, BMI, and AGE Accumulation

In this study, non-invasive skin AGE levels were detected in community populations to observe possible influencing factors. The first is the detection of biochemical indicators, including SBP, TC, TG, HDL, BMI, HbA1c, FBG, and AGE. Normally, the generation of AGEs goes through a slow glycosylation stage, and hyperglycemia, hyperlipidemia, or oxidative stress can accelerate the accumulation of AGEs in the body (32). Obesity and what is referred to as insulin resistance (IR) syndromes, including hypertension, glucose intolerance, hyperinsulinemia, and hyperlipidemia, are common factors (33). In this study, multivariate logistic regression analysis showed that AGEs were associated with both T2DM and prediabetes risk. Therefore, it could be speculated that elevated levels of AGEs in the skin could be a warning sign of T2DM, and high serum glycated hemoglobin A1c (HbA1c) can accelerate the formation of AGEs and hydroxymethyl lysine. Even if HbA1c is well controlled, high levels of AGEs can also increase the risk of diabetic microvascular complications and form the “metabolic memory” effect of hyperglycemia (34). Patients with type 1 T2DM were followed up for 7 years. It was found that AGE fluorescence spectrum detection of skin could predict the occurrence of major adverse cardiovascular events in patients with type 1 T2DM; the higher the intensity of AGE fluorescence in skin, the higher the probability of myocardial infarction, stroke, lipid metabolism index, lower-limb paraplegia, or revascularization surgery in patients with type 1 T2DM (35). In addition, a previous study proposed that AGE was also connected to the pathophysiology of obesity (36). Another study has reported that obese adults (average body mass index [BMI] 33.2 kg/m^2^) exhibited significantly higher-circulating AGEs compared to overweight participants (average BMI 26.3 kg/m^2^) (37). Serum AGEs were found to correlate with triglyceride levels, and those with the highest AGEs had the most adverse lipid profiles (38). Another study demonstrated that AGE was also associated with kidney function (39). Similarly, age and gender differences were also verified to be associated with AGE. Serum AGEs have been reported to be in the range of 8.5 ± 0.9 units/ml in men less than age 45, 9.9 ± 1.5 in men older than 60, 7.9 ± 0.7 in women less than age 45, and 10.7 ± 1.1 in women older than 60 in a study of 172 healthy individuals (40).

### The Moderating Role of BMI, Gender Between Health Risk Factors, and AGE Accumulation

Our research suggests that participants, especially those with a higher BMI and higher health risk factors, seem to be a vulnerable group for AGE accumulation. From a developmental disease perspective, obesity interacts with health risk factors, influencing the development of AGE accumulation. BMI, as a moderating variable, has gender-specific effects on the association between HRFs and AGE accumulation. The quality of gender moderates the relationship between BMI and AGE accumulation. As we all know, HbA1c and obesity were all associated with AGE accumulation; AGEs may play a more prominent role in the health of obese individuals, so obesity may be one of the health risk factors and pathophysiology mechanisms leading to AGE accumulation in health risk factors (39, 41). Moreover, we also added gender: according to the diathesis-stress model, females are more vulnerable to the higher effects of HRFs in a higher BMI (obesity), consequently leading to greater female-to-AGE accumulation. Although components demonstrating this inverse relationship included body mass index, TG, HbA1c, and insulin resistance, which were all inversely linked with RAGEs (receptor for AGEs) in both diabetic and non-diabetic participants (42), this also verifies that the indicators selected in this study are reasonable. Another reason was that AGEs increase with increasing BMI whereas the link is opposite for sRAGE; sRAGE may reflect tissue RAGE expression and that sRAGE may increase along with AGE in order to mount a counter-defense (43).

### Study the Theoretical and Clinical Significance of AGE

Fluorescent intensity of AGEs in the skin can reflect the longer time of blood glucose control, which may be one of the indicators to predict the chronic complications and mortality of T2DM (44). Previous studies have shown that the interaction between AGEs and its receptor RAGE causes oxidative stress, inflammation, and fibrosis, which can lead to endothelial dysfunction, atherosclerosis, vascular stiffness, progressive changes in renal structure, and impaired renal function (32, 45, 46). Therefore, non-invasive skin detection for AGEs is of great significance for the prevention and monitoring of T2DM and its complications. Venous blood FPG, OGTT, and glycated hemoglobin are important indicators for the screening and diagnosis of T2DM, but there are some problems, such as invasiveness, high cost, and long time to transport samples (47, 48). A large number of foreign studies have reported that skin autofluorescence can be used for T2DM screening, and the sensitivity of the diagnosis of T2DM is higher than FPG or HbA1c (48–50). Moreover, these non-invasive measurements of AGE accumulation may be considered as promising biomarkers of late diabetic complications.

### Limitation

The following are the study’s limitation: a large number of foreign studies have reported that skin autofluorescence (SAF) can be used for T2DM screening, and the sensitivity of the diagnosis of T2DM is higher than FPG or HbA1c. The authors did not investigate the subjects’ family history of T2DM, dietary habits, etc., which may affect the results of the study (39). In addition, Noordzij reported that skin autofluorescence detection may have some limitations, such as skin pigmentation, use of cream and sunscreen, and extreme congestion or vasoconstriction, which may affect the measurement results (51).

## Conclusion

This study was based on existing literature documenting possible associations between AGE accumulation, BMI, and health risk factors in a Chinese cultural context. Health risk factors should be taken into account when designing interventions for AGE-cumulative primary prevention in participants, especially in women. Reducing the incidence of health risk factors can significantly improve BMI at lower levels. In addition, strengthening a lower BMI can prevent or reduce AGE accumulation, especially in women. The study also suggests that Chinese participants can use weight control therapy as an adjunct to adjusting BMI when treating adolescents with higher HRFs. In addition, with BMI as a moderating variable, the relationship between HRF and AGE accumulation is different between sexes. The findings point to the necessity to focus on normal BMI and use gender-specific approaches to address AGE accumulation in patients with a history of higher HRFs.

In general, non-invasive skin detection of AGEs has a certain application value for the assessment of T2DM risk but may not be fully applicable for early screening of T2DM. Much has yet to be done in AGE research. More studies are needed to confirm this non-invasive technique as a new T2DM screening tool to improve the diagnosis rate of T2DM.

## Data Availability Statement

The raw data supporting the conclusions of this article will be made available by the authors, without undue reservation.

## Ethics Statement

The studies involving human participants were reviewed and approved by Ethics Committee of the First Affiliated Hospital of Anhui Medical University Ethics Committee (Ethics Approval No.: PJ2019-09-05). The patients/participants provided their written informed consent to participate in this study. Written informed consent was obtained from the individual(s) for the publication of any potentially identifiable images or data included in this article.

## Author Contributions

QZ constructed the study design. FD and HH recruited the participants. YZ, FD, and TJ were involved in the statistical analysis. QZ was responsible for the critical revision of the manuscript. YZ, TJ, and CL edited and revised the manuscript. YZ and CL prepared and drafted the manuscript. All the authors who contributed to the manuscript gave their approval for its submission. The work presented here has not been published previously and is not being considered for publication elsewhere. All authors contributed to the article and approved the submitted version.

## Funding

The author(s) disclosed receipt of the following financial support for the research, authorship, and/or publication of this article: funding for the project was provided by National Natural Science Foundation of China (81970703).

## Conflict of Interest

The authors declare that the research was conducted in the absence of any commercial or financial relationships that could be construed as a potential conflict of interest.

## Publisher’s Note

All claims expressed in this article are solely those of the authors and do not necessarily represent those of their affiliated organizations, or those of the publisher, the editors and the reviewers. Any product that may be evaluated in this article, or claim that may be made by its manufacturer, is not guaranteed or endorsed by the publisher.
